# Supplementary material for: Health seeking behaviors of nurses diagnosed with hypertension and providing health care in resource-constrained setting in a rural part of Northern Ghana: A qualitative study
Source: PLoS One. 2026 Feb 11;21(2):e0342406. doi: 10.1371/journal.pone.0342406 (PMC12893536; doi:10.1371/journal.pone.0342406)
Supplement: S1 File — (DOCX) [file pone.0342406.s001.docx]

S1

**Appendix 1: Interview Guide**

The interview guide was developed using the constructs of the Health Belief Model (HBM). Each section of the guide reflects HBM domains: questions on risk perception address perceived susceptibility and severity; questions on coping and treatment reflect perceived benefits and barriers; while items on workplace culture and social support capture cues to action and self-efficacy. This theoretical grounding ensures that the guide elicits data aligned with the study’s objectives.

**1. Demographic Information**

- Age:
- Gender:
- Highest Level of Education:
- Years of Experience in Nursing:
- Current Job Title:

**2. Health Awareness**

***Understanding of Hypertension****:*

How would you describe your understanding of hypertension and its implications for nursing professionals?

***Information Sources****:*

What sources do you rely on for information regarding hypertension management?

**3. Perceptions of Severity**

***Severity Assessment****:*

In your view, how severe is hypertension among nurses, and what factors contribute to this perception?

***Impact on Performance****:*

Have you ever felt that your health condition impacts your performance or well-being at work? If so, please elaborate.

**4. Management Practices**

***Personal Strategies****:*

What specific strategies do you personally employ to manage your hypertension?

***Challenges in Management****:*

What challenges do you encounter in effectively managing hypertension while balancing your nursing responsibilities?

**5. Cultural Influences**

***Organizational Culture****:*

How does the organizational culture in your workplace influence your approach to managing hypertension?

***Perception of Stigma****:*

Do you perceive any stigma associated with discussing health issues, particularly hypertension, among nursing staff? Please explain.

**6. Social Support**

***Colleague and Supervisor Support****:*

In what ways do your colleagues and supervisors support you in managing your hypertension?

***Family Contribution****:* How does your family contribute to your health management efforts?

**7. Suggestions for Improvement**

***Resources and Support Systems****:*

What additional resources or support systems would enhance your ability to manage hypertension effectively?

***Organizational Improvements****:*

How can healthcare organizations improve support for nurses in maintaining their health and well-being, particularly concerning hypertension?

What else do you want to tell me regarding the issue we are discussing?

Thank you.
