# Supplementary material for: Health seeking behaviors of nurses diagnosed with hypertension and providing health care in resource-constrained setting in a rural part of Northern Ghana: A qualitative study
Source: PLoS One. 2026 Feb 11;21(2):e0342406. doi: 10.1371/journal.pone.0342406 (PMC12893536; doi:10.1371/journal.pone.0342406)
Supplement: Attachment — Submitted filename: Response to reviewers .pdf [file pone.0342406.s006.pdf]

University of Ghana
College of Health Sciences
School of Nursing and Midwifery
Department of Adult Health

26th DECEMBER, 2025

The Editor
PLOS ONE

Dear Sir/Madam,

Response to review

General comments of authors

We have addressed all the comments of the editor and reviewers as suggested and we hope the revised manuscript meets the standards for publications.

Comments of the Editor

PONE-D-25-57892

Health seeking behaviors of nurses diagnosed with hypertension and providing health care in resource-constrained setting in a rural part of Northern Ghana: A qualitative study

PLOS One

Dear Dr. Konlan,

Dear Dr. Konlan,

Thank you for submitting your manuscript to PLOS ONE. After careful consideration, we feel that it has merit but does not fully meet PLOS ONE’s publication criteria as it currently stands. Therefore, we invite you to submit a revised version of the manuscript that addresses the points raised during the review process.

Please submit your revised manuscript by Feb 09 2026 11:59PM. If you will need more time than this to complete your revisions, please reply to this message or contact the journal office at plosone@plos.org. When you're ready to submit your revision, log on to https://www.editorialmanager.com/pone/ and select the 'Submissions Needing Revision' folder to locate your manuscript file.

Please include the following items when submitting your revised manuscript:

- A letter that responds to each point raised by the academic editor and reviewer(s). You should upload this letter as a separate file labeled 'Response to Reviewers'.

- A marked-up copy of your manuscript that highlights changes made to the original version. You should upload this as a separate file labeled 'Revised Manuscript with Track Changes'.
- An unmarked version of your revised paper without tracked changes. You should upload this as a separate file labeled 'Manuscript'.

If you would like to make changes to your financial disclosure, please include your updated statement in your cover letter. Guidelines for resubmitting your figure files are available below the reviewer comments at the end of this letter.

If applicable, we recommend that you deposit your laboratory protocols in protocols.io to enhance the reproducibility of your results. Protocols.io assigns your protocol its own identifier (DOI) so that it can be cited independently in the future. For instructions see: https://journals.plos.org/plosone/s/submission-guidelines#loc-laboratory-protocols. Additionally, PLOS ONE offers an option for publishing peer-reviewed Lab Protocol articles, which describe protocols hosted on protocols.io. Read more information on sharing protocols at https://plos.org/protocols?utm_medium=editorial-email&utm_source=authorletters&utm_campaign=protocols.

We look forward to receiving your revised manuscript.

Kind regards,

Javier Fagundo-Rivera, PhD
Academic Editor
PLOS One

Journal Requirements:

When submitting your revision, we need you to address these additional requirements.

1.Please ensure that your manuscript meets PLOS ONE's style requirements, including those for file naming. The PLOS ONE style templates can be found at

https://journals.plos.org/plosone/s/file?id=wjVg/PLOSOne_formatting_sample_main_body.pdf and

https://journals.plos.org/plosone/s/file?id=ba62/PLOSOne_formatting_sample_title_authors_affiliations.pdf

2. We note that your Data Availability Statement is currently as follows: All relevant data are within the manuscript and in Supporting Information files.

Please confirm at this time whether or not your submission contains all raw data required to replicate the results of your study. Authors must share the “minimal data set” for their submission. PLOS defines the minimal data set to consist of the data required to replicate all study findings reported in the article, as well as related metadata and methods (https://journals.plos.org/plosone/s/data-availability#loc-minimal-data-set-definition).

For example, authors should submit the following data:

- The values behind the means, standard deviations and other measures reported;

- The values used to build graphs;

- The points extracted from images for analysis.

Authors do not need to submit their entire data set if only a portion of the data was used in the reported study.

If your submission does not contain these data, please either upload them as Supporting Information files or deposit them to a stable, public repository and provide us with the relevant URLs, DOIs, or accession numbers. For a list of recommended repositories, please see https://journals.plos.org/plosone/s/recommended-repositories.

If there are ethical or legal restrictions on sharing a de-identified data set, please explain them in detail (e.g., data contain potentially sensitive information, data are owned by a third-party organization, etc.) and who has imposed them (e.g., an ethics committee). Please also provide contact information for a data access committee, ethics committee, or other institutional body to which data requests may be sent. If data are owned by a third party, please indicate how others may request data access.

3. Your ethics statement should only appear in the Methods section of your manuscript. If your ethics statement is written in any section besides the Methods, please move it to the Methods section and delete it from any other section. Please ensure that your ethics statement is included in your manuscript, as the ethics statement entered into the online submission form will not be published alongside your manuscript.

4. Please include captions for your Supporting Information files at the end of your manuscript, and update any in-text citations to match accordingly. Please see our Supporting Information guidelines for more information: http://journals.plos.org/plosone/s/supporting-information.

5. If the reviewer comments include a recommendation to cite specific previously published works, please review and evaluate these publications to determine whether they are relevant and should be cited. There is no requirement to cite these works unless the editor has indicated otherwise.

Additional Editor Comments:

Dear Authors,

Thank you for your work and for your interest in PLOS ONE as a venue to disseminate your research.

Three reviewers have evaluated your manuscript. A major revision is required, addressing the comments from all three reviewers.

Please revise your manuscript accordingly and submit a detailed response-to-reviewers letter indicating how you have addressed each of the reviewers' comments.

Kind regards,

[Note: HTML markup is below. Please do not edit.]

Authors' Response to Comments of editor

General Response to editor's comments

We are grateful for the comments of the editor and have addressed all the concerns and comments of the editor.

Authors' response to Journal Requirements:

1. We have ensured that our manuscript meets PLOS ONE's style requirements, including those for file naming.

2. We have ensured that our ethics statement appeared only in the Methods section of our manuscript. This is found on page 11 of the revised manuscript.

3. We have stated on page 11 that all the participants gave consent for interview transcript to be published.

4. We have removed all personal identification information in the revised manuscript

5. This was not applicable to our manuscript.

Additional Editor Comments:

We have addressed the comments of the editor in the revised manuscript.

REVIEWER 1 COMMENTS

Reviewer #1: SEE DOCUMENT ATTACHED

This study, titled "Health seeking behaviors of nurses diagnosed with hypertension and providing health care in resource-constrained setting in a rural part of Northern Ghana: A qualitative study," explores the personal health management strategies and associated challenges faced by nurses living with hypertension in a high-stress, resource-constrained environment in Ghana. The manuscript provides valuable insights into the persistent gap between high professional knowledge and poor health-seeking behavior, attributing this divergence primarily to organizational and systemic barriers. I reviewed the study following PLOS ONE criteria which I now submit.

Authors' response to reviewer 1

We are grateful for the comments of the reviewer. We have addressed the grammatical errors and we are grateful for the favourable comments of the reviewer in the attached filed.

Reviewer 2 comments

Reviewer #2:

I recommend major revision: (1) sample/ID inconsistency (n=12 but quotes labeled "Nurse 13"); (2) clarify recruitment, voluntariness, and privacy (management involvement; interview location); (3) align the Data Availability statement with PLOS requirements; (4) correct the Limitations section.

Authors' responses to comments of reviewer 2

1. We have ensured the sample/ID are consistent and removed the error of nurse 13 found in the manuscript. This is found on page 6 on the sample size and also in the results on pages 12 to 28 of the revised manuscript.
2. We have clarified the recruitment, voluntariness, and privacy (management involvement; interview location). This is found on page 8 of the revised manuscript on selection of participants and data collection.
3. We have aligned data availability with PLOS requirements as suggested by the reviewer.
4. We have worked on the limitations section of the revised manuscript. This is found on page 13 of the revised manuscript.

Reviewer 3 comments

Reviewer #3: SEE DOCUMENT ATTACHED

The manuscript is scientifically sound and backed by data. The authors need to correct grammar mistakes and try not to mix discussion with results, otherwise the manuscript reflects good scientific

rigor and the conclusions and recommendations tie up well with the findings of the study. With minor corrections, it is a good manuscript for publication.

Authors' responses to comments of reviewer 3

We are grateful for the comments of the reviewer.

We have worked on the grammatical mistakes and ensured that the discussion is separated from the results in the revised manuscript.

We have addressed all the concerns of the reviewer.

We hope the revised manuscript will meet the standards for publication.

Thank you

Yours sincerely,
Dr. Kennedy Dodam Konlan
(Corresponding author)
