# Supplementary material for: Health seeking behaviors of nurses diagnosed with hypertension and providing health care in resource-constrained setting in a rural part of Northern Ghana: A qualitative study
Source: PLoS One. 2026 Feb 11;21(2):e0342406. doi: 10.1371/journal.pone.0342406 (PMC12893536; doi:10.1371/journal.pone.0342406)
Supplement: Attachment — Submitted filename: MGB Revision of PONE-D-25-57892.docx [file pone.0342406.s003.docx]

**Revision of PONE-D-25-57892**

This study, titled "Health seeking behaviors of nurses diagnosed with hypertension and providing health care in resource-constrained setting in a rural part of Northern Ghana: A qualitative study," explores the personal health management strategies and associated challenges faced by nurses living with hypertension in a high-stress, resource-constrained environment in Ghana. The manuscript provides valuable insights into the persistent gap between high professional knowledge and poor health-seeking behavior, attributing this divergence primarily to organizational and systemic barriers. I reviewed the study following PLOS ONE criteria which I now submit:

**Criterion 1: The study presents the results of original research**

The manuscript fulfills this criterion as it reports the findings of an original qualitative research project. The aim was specifically to explore the health-seeking behaviors of nurses with hypertension in a resource-constrained rural setting in Northern Ghana, addressing a stated limitation in existing evidence regarding this high-stress population in Ghana. The study employed a qualitative, exploratory-descriptive design to gather comprehensive information on the experiences and contextual factors influencing the health of twelve purposively sampled nurses.

**Criterion 2: Results reported have not been published elsewhere**

The results reported appear to be original and unpublished. The authors have declared that the data concerning rising hypertension trends among nurses at the study site, cited in the introduction, were derived from unpublished internal reports, suggesting the primary data presented here are novel. Furthermore, the authors confirmed that they received no specific funding for the work and declared no competing interests, standardizing the submission process for original work.

**Criterion 3: Experiments, statistics, and analyses are performed to a high standard and are described in sufficient detail**

The methodology for this qualitative study is described in detail and adheres to high standards of rigor. The study utilized a qualitative, exploratory-descriptive design employing purposive sampling to recruit twelve nurses from the Kintampo Municipal Hospital’s Employee Wellness Clinic. The sample size was determined by data saturation, which was achieved after the twelfth interview, following the observation of data redundancy after the ninth. Data were collected via in-depth, semi-structured interviews (35-45 minutes long) conducted in English, audio-taped, and transcribed verbatim. The data analysis employed thematic analysis guided by Braun and Clarke's framework and supported by NVivo 14.0 software. Rigor was addressed through member checking, prolonged engagement, peer debriefing, detailed audit trails, and consistent procedures. Furthermore, triangulation was achieved through multiple data sources, investigator involvement in coding, and interpretation using multiple theories, including the Health Belief Model, which served as the analytical framework.

**Criterion 4: Conclusions are supported by the data and presented appropriately**

The study's conclusion, which posits that a significant gap between nurses' high knowledge and their poor health-seeking actions is driven by organizational and systemic barriers, is well-supported by the derived results. The analysis yielded nine major themes, including "Barriers to Healthcare Access and Management," highlighting overwhelming workload, scheduling constraints, financial limitations, and lack of institutional support. The discussion effectively uses the Health Belief Model (HBM) to frame the findings, showing that high perceived threat (severity/knowledge) is overcome by substantial perceived barriers (stigma, professional self-reliance, workload), which inhibits action. Recommendations, such as prioritizing structured, low-cost workplace wellness programs, flexible scheduling, and anti-stigma campaigns, directly address the systemic barriers identified in the data.

**Criterion 5: The article is intelligible and written in standard English**

The manuscript is written in clear, concise, and academically appropriate English throughout the Abstract, Methods, Results, and Discussion sections. The language is articulate and the presentation of complex methodological concepts, such as triangulation and HBM application, is clear. The interviews themselves were conducted in English, which is noted as the official language of communication in Ghana.

**Criterion 6: The research meets ethical standards and is ethically sound**

The research strictly adhered to high ethical standards. The authors confirmed compliance with the Declaration of Helsinki regarding data collection. The study received approval from the Kintampo Research Center’s Scientific Review Committee (Protocol Number: khrc/adm/14/2025-10) and institutional approval was sought prior to recruitment. Written informed consent was obtained from all participants after the study purpose was explained. Confidentiality, privacy, and anonymity were maintained throughout data collection and analysis by assigning unique codes to responses and securely storing, then deleting, the audio recordings upon completion of transcription.

**Criterion 7: The article adheres to reporting guidelines and community data availability standards**

The authors have complied with essential reporting requirements. They explicitly stated that no specific funding was received for the work and declared that no competing interests exist. Regarding data availability, the authors confirmed that all data underlying the findings are fully available without restriction. The Data Availability Statement confirms that "All relevant data are within the manuscript and its Supporting Information files". Furthermore, the ethical clearance letter and the interview guide are provided as Supporting Information files.
