# Supplementary material for: Health seeking behaviors of nurses diagnosed with hypertension and providing health care in resource-constrained setting in a rural part of Northern Ghana: A qualitative study
Source: PLoS One. 2026 Feb 11;21(2):e0342406. doi: 10.1371/journal.pone.0342406 (PMC12893536; doi:10.1371/journal.pone.0342406)
Supplement: Attachment — Submitted filename: renamed_18dc9.docx [file pone.0342406.s005.docx]

**Revision of ‘PONE-D-25-57892R1’**

This revised manuscript demonstrates substantial improvement in clarity, structure, and methodological transparency. The authors have engaged seriously with previous reviewer comments and have strengthened the manuscript in ways that make it both analytically rigorous and highly relevant for publication. The study now presents a coherent, well-documented, and ethically sound contribution that is appropriate for the scope of PLOS ONE. Considering the revisions made and the overall quality of the manuscript, I recommend acceptance.

**1. Originality and Contribution to Knowledge**

The study offers a valuable and underexplored perspective on how nurses diagnosed with hypertension manage their own health in a rural, resource-constrained environment. By situating individual behavior within organizational demand, institutional culture, and systemic barriers, the manuscript contributes insights that are both conceptually meaningful and practically relevant. The work clearly meets PLOS ONE expectations regarding original research that adds to the existing literature.

**2. Prior Publication and Redundancy**

The authors clearly state that the results have not been previously published, and there is no evidence of duplicate or redundant submission. The manuscript satisfies the journal’s originality requirement.

**3. Methodological Rigor, Transparency, and Reporting**

The revised manuscript shows clear improvement in methodological articulation. The description of sampling, recruitment processes, voluntariness, confidentiality, and data management is now sufficiently detailed and transparent. The explanation of thematic analysis, including how NVivo supported data handling, is well presented. The manuscript also provides an explicit account of trustworthiness strategies, including credibility, dependability, reflexivity, and triangulation, thereby aligning well with qualitative research standards.

Ethical procedures, including approvals, informed consent, privacy protections, and protocols for participants experiencing distress, are described thoroughly and appropriately located within the Methods section. Overall, methodological rigor and transparency are satisfactory.

**4. Alignment of Conclusions with Findings**

The conclusions are well justified by the data. The analysis convincingly demonstrates that structural and organizational barriers, rather than lack of knowledge, shape nurses’ largely reactive approach to health-seeking. The recommendations provided are realistic, grounded in findings, and sensitive to the context of rural health systems. The discussion maintains appropriate alignment between evidence and interpretation.

**5. Clarity of Writing and Use of English**

The prose is clearer and more polished in the revised version. The narrative is coherent, academically appropriate, and readable. Minor stylistic imperfections remain, but they do not detract from comprehension or overall presentation.

**6. Ethical Standards and Approvals**

Ethical oversight, consent procedures, confidentiality measures, and institutional permissions are clearly documented. The manuscript adheres to ethical standards consistent with PLOS ONE policy.

**7. Reporting Standards and Data Availability**

The revised manuscript aligns with PLOS ONE reporting expectations. The Data Availability Statement is appropriate and supporting files enhance transparency. The level of information shared is sufficient for readers to follow the analytic process and understand the evidence supporting the conclusions.
